# Prediction of antipsychotic medication inception in antipsychotic-naive youth at clinical high risk for psychosis

**DOI:** 10.1017/S0033291725101372

**Published:** 2025-08-22

**Authors:** Hesham Mukhtar, Dolores Zhou, Emily A. Farina, Abhishek Saxena, John Cahill, Jean Addington, Carrie E. Bearden, Kristen S. Cadenhead, Tyrone D. Cannon, Barbara A. Cornblatt, Matcheri S. Keshwan, Daniel H. Mathalon, Diana O. Perkins, William S. Stone, Youngsun T. Cho, Albert R. Powers, Elaine F. Walker, Scott W. Woods

**Affiliations:** 1 Department of Psychiatry, Yale University School of Medicine and the Connecticut Mental Health Center, New Haven, CT, USA; 2 College of Arts & Sciences, Emory University, Atlanta, GA, USA; 3 Department of Psychiatry, Hotchkiss Brain Institute, University of Calgary, Calgary, AB, Canada; 4 Department of Psychiatry and Biobehavioral Sciences, University of California Los Angeles, Los Angeles, CA, USA; 5 Department of Psychiatry, University of California San Diego, CA, USA; 6 Department of Psychology, Yale University, New Haven, CT, USA; 7 Department of Psychiatry, Zucker Hillside Hospital, Long Island, NY, USA; 8 Department of Psychiatry, Harvard Medical School at Beth Israel Deaconess Medical Center and Massachusetts General Hospital, Boston, MA, USA; 9 Department of Psychiatry, UCSF, and SF VA Medical Center, San Francisco, CA, USA; 10 Department of Psychiatry, University of North Carolina, Chapel Hill, NC, USA; 11 Child Study Center, Yale University School of Medicine, New Haven, CT, USA; 12 Departments of Psychology and Psychiatry, Emory University, Atlanta, GA, USA

**Keywords:** Clinical high risk, antipsychotics, major depression, AP inception

## Abstract

**Background:**

Antipsychotic (AP) medication in individuals at clinical high risk for psychosis (CHR-P) is not routinely recommended by clinical guidelines but is commonly prescribed. Since little is known about the predictors of AP inception in CHR-P, we analyzed data from two observational cohorts.

**Methods:**

To avoid baseline predictors being confounded by previous treatment, participants were selected for analysis from the 764 participants at CHR-P enrolled in NAPLS-2 and the 710 enrolled in NAPLS-3 by excluding those with lifetime histories of AP use. Baseline clinical variables available in both studies were employed as predictors of subsequent AP inception over the next 6 months in univariable and multivariable analyses.

**Results:**

Preliminary analyses indicated no important effects of sample. The final combined population included 79 AP inception participants and 580 participants who did not have AP inception. The AP medications most commonly prescribed were risperidone, aripiprazole, and quetiapine. Univariable analyses identified seven significant predictors of AP inception. The final logistic regression model including these variables was highly significant (χ^2^ = 36.53, df = 7, *p* = <0.001). Three variables (current *major depression*, fewer education years, and current benzodiazepine use) emerged as significant independent predictors of AP inception.

**Conclusion:**

This study is the first to determine baseline characteristics that predict subsequent AP initiation in CHR-P. Some AP use in CHR-P appears to be intended as augmentation of antidepressant treatment for comorbid major depression. Some prescribers may not have detected the attenuated positive symptoms characteristic of CHR-P since their severity did not significantly predict AP inception.

## Introduction

The clinical high risk syndrome for psychosis (CHR-P) offers a paradigm that identifies a clinically important population (Salazar, Catalan, & Fusar-Poli, 2020) and has made important contributions to early detection and intervention for psychosis. CHR-P is a common condition, as evidenced by systematic review findings that CHR-P services provide care for over 28 million people across five continents (Estradé et al., 2022) and by a meta-analysis showing a CHR-P prevalence in the general youth population of 1.7% and 19.2% of youth presenting for psychiatric care (de Pablo, Woods, Drymonitou, de Diego, & Fusar-Poli, 2021). CHR-P is associated with a 20% probability of developing psychosis at 2 years, which increases over the long term peaking to 35% at 10 years (de Pablo et al., 2021), and at least 78% of patients with first episode psychosis go through a CHR-P phase before becoming psychotic (Benrimoh et al., 2024). Early detection enables the provision of targeted therapies that may prevent or mitigate the onset of full-blown psychotic disorders.

The use of antipsychotics in individuals at CHR-P for psychosis presents a complex dilemma in psychiatric treatment. Treatment guidelines generally do not recommend antipsychotic use as first-line treatment in CHR-P patients (Addington, Addington, Abidi, Raedler, & Remington, 2017; Poletti, Pelizza, Preti, & Raballo, 2024; Schmidt et al., 2015) due to concerns about side effects and the uncertain benefit in delaying or preventing psychosis. Nevertheless, these medications are still frequently prescribed in this population (Raballo, Poletti, & Preti, 2020; Woods et al., 2013), in some cohorts to >50% of participants (Pelizza et al., 2024; Zhang et al., 2022).

Despite the clinical importance of CHR-P, and the disconnect between clinical guidelines and clinical practice, little is known about the predictors of AP inception in CHR-P aside from reports from two cohorts where prediction appears to have been confounded by AP start before the baseline assessment of predictors. In one previous report (Zeng et al., 2022), initially AP-naive CHR-P patients from the 2016–2021 Shanghai cohort (total *N* = 717) had their first visit with clinicians where decisions regarding AP prescription were made. Baseline study assessments were collected after the clinical visit, but the time interval between the prescription and the baseline assessment was not reported, leaving open the possibility that the baseline assessments that were tested for correlation with AP treatment had been affected by that treatment. In another report from the same cohort (total *N* = 600), the AP medication was described as ‘ongoing at enrollment’ (Zhang et al., 2022). A recent study from Parma in northern Italy also reported on correlations between baseline AP use and baseline severity (Pelizza et al., 2024) in a cohort that permitted previous AP use at baseline when that use was for no more than 4 weeks before assessment.

Since only two previous studies exist and each presents methodologic limitations, we analyzed data from two observational cohorts to determine the predictors of antipsychotic medication inception in participants at CHR-P for psychosis. The NAPLS-2 and NAPLS-3 studies offer large samples with well-characterized lifetime histories of psychotropic medication use. In order to minimize confounding factors for the prediction of AP inception, the design applied to the samples required inclusion in analysis of only those participants who never received AP in their lifetime prior to baseline assessment. To minimize the potential for intercurrent events between baseline and subsequent AP inception that could confound baseline prediction, the time frame of 6 months after baseline was selected for categorizing participants who had an AP start versus those who did not.

## Methods

Participants were included according to the PICO (population, intervention, comparison, outcome) criteria. The population consisted of participants at clinical high risk for psychosis, ages 12 to 35 years, from the NAPLS-2 (Addington et al., 2012) and NAPLS-3 (Addington et al., 2022) cohorts. There was no intervention or comparison group. The outcome was first lifetime use of antipsychotic medication within 6 months after study enrollment. Both NAPLS-2 and -3 cohorts recruited participants who met the criteria of prodromal syndromes (COPS) articulated in the structured interview for psychosis-risk syndromes (SIPS) (Miller et al., 2002). All of the demographic (age, gender, Caucasian, Latino, education years, household income), diagnostic (SIPS APSS, SIPS BIPS, SIPS GRD, current major depression, current bipolar disorder, current anxiety disorder), functioning (current GAF, change in GAF, current GFS, change in GFS, current GFR, change in GFR), symptoms (SOPS total, SOPS positive, SOPS negative, SOPS disorganized, SOPS general, CDSS total), and medication variables (current use of antidepressant, mood stabilizer, psychostimulant, benzodiazepines [BZ], non-BZ anxiolytic, any psychotropic) common to both cohorts were used for the prediction analysis. Major depression, anxiety disorder, and bipolar disorder were assessed using the Structured Clinical Interview for *DSM* (SCID-IV).

### Participants

**Figure 1.**
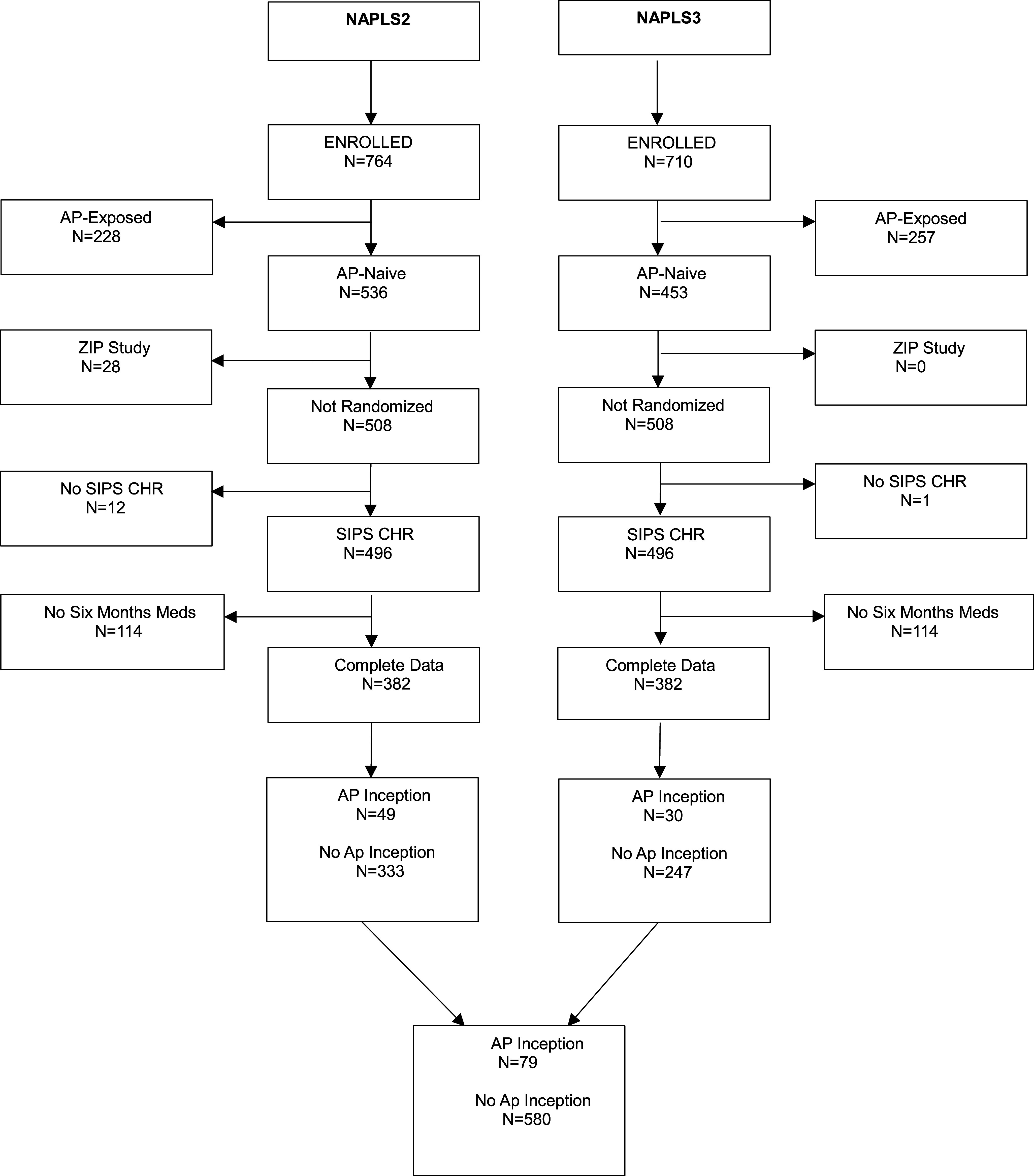
Consort diagram. CONSORT diagram for CHR-P participants enrolled in NAPLS-2 and NAPLS-3 samples. For the analysis only those participants were included who had no lifetime antipsychotic use at baseline. NAPLS-2 included some nonrandomized clinical trial participants which were also removed. In addition, only those participants were included who met the SIPS CHR-P criteria and had complete 6 months medications data. Participants were finally divided in those who had an AP inception during the first 6 months and those who had no AP inception during the first 6 months.

Figure 1 shows how participants were selected for analysis from the 764 CHR-P enrolled in NAPLS-2 (Addington et al., 2012) and the 710 enrolled in NAPLS-3 (Addington et al., 2022). In order to avoid the potential confounding effects of previous AP medication on the baseline predictors, participants with lifetime histories of previous antipsychotic use were excluded. Because patients randomized in a ziprasidone clinical trial embedded in NAPLS-2 started AP for different reasons from the remaining participants who started for clinical reasons, the randomized participants were also removed. The analysis was limited to those meeting SIPS CHR-P criteria (Miller et al., 2002) and those who had complete medication data for 6 months. AP inception participants were those who had AP start for the first time in their lives while qualifying for CHR-P during the first 6 months, and no AP inception participants were those who had documented absence of AP start while CHR-P during the first 6 months. Cases were included whether or not they subsequently converted to psychosis. Participants who began AP during the first 6 months only after conversion did not experience AP inception while CHR-P and were counted as *no AP inception* participants. To evaluate possible bias due to missing medication data, baseline data were compared between the participants with complete medication data and those without complete data using the *T* test and chi-square test (Table S1 data supplement).

### Prediction analyses

Analyses of AP inception predictors employed baseline clinical variables from the NAPLS-2 and -3 cohorts described earlier, including demographic, diagnostic, medication, symptom, and functioning measures (Table 1).

**Table 1.** Univariable description of NAPLS-2 + NAPLS-3 CHR-P samples who were antipsychotic-naive at baseline (*N* = 659) by subsequent antipsychotic inception

Measure	AP inception in first 6 months (*N* = 79)	No AP inception in first 6 months (*n* = 580)	Odds ratio	95% Confidence interval		*p*
				95% LB	95% UB	
Age	17.82 ± 3.90	18.58 ± 4.30	0.957	0.902	1.105	0.124
Male gender	40(50.6%)	325(56%)	0.802	0.501	1.285	0.376
Caucasian	48(60.8%)	314(54.1%)	1.307	0.809	2.114	0.268
Latino^a^	17(21.5%)	107(18.5%)	1.224	0.687	2.180	0.484
Household income^b^	4.52 ± 2.01	4.76 ± 1.89	0.938	0.831	1.059	0.320
Education years^c^	10.70 ± 3.04	11.40 ± 3.27	0.936	0.871	1.005	0.038
SIPS APSS	78 (98.7%)	561 (96.7%)	2.869	0.376	21.885	0.170
SIPS BIPS	3 (3.8%)	8 (1.4%)	2.649	0.678	10.347	0.104
SIPS GRD	7 (8.9%)	36 (6.2%)	1.401	0.594	3.302	0.449
Current major depression	49 (62.0%)	226 (39.0%)	2.566	1.581	4.164	0.001
Current bipolar disorder^b^	2 (2.5%)	34 (5.9%)	0.000			0.001
Current anxiety disorder	51 (64.6%)	302 (52.1%)	1.744	1.064	2.860	0.032
Antidepressant	23 (29.1%)	123 (21.2%)	1.555	0.919	2.634	0.104
Mood stabilizer	1 (1.3%)	8 (1.4%)	0.921	0.114	7.469	0.563
Psychostimulant	6 (7.6%)	32 (5.5%)	1.394	0.563	3.450	0.488
Benzodiazepine	11 (13.9%)	22 (3.8%)	4.050	1.880	8.726	0.002
NonBZ anxiolytic	0	3 (0.5%)	0.000			0.001
Any psychotropic	33 (41.8%)	155 (26.7%)	1.992	1.227	3.234	0.006
SOPS total^d^	40.19 ± 10.76	37.45 ± 12.38	1.019	1.000	1.040	0.033
SOPS positive^b^	12.87 ± 2.81	12.31 ± 3.70	1.051	0.983	1.123	0.063
SOPS negative^e^	12.11 ± 5.21	11.44 ± 6.13	Noninterpretable			
SOPS disorganized^c^	4.94 ± 2.84	4.94 ± 3.08	1.001	0.927	1.082	0.981
SOPS general^e^	10.27 ± 4.35	8.82 ± 4.21	1.086	1.026	1.150	0.005
CDSS total^f^	7.74 ± 5.18	5.58 ± 4.45	1.100	1.048	1.154	0.001
Current GAF^g^	48.92 ± 9.45	51.21 ± 11.33	0.982	0.961	1.003	0.051
Current GFS^b^	6.37 ± 1.57	6.42 ± 1.52	0.980	0.840	1.143	0.804
Current GFR^c^	6.39 ± 2.27	6.30 ± 2.15	Noninterpretable			
Change GAF^h^	−9.96 ± 12.23	−8.80 ± 10.89	0.992	0.971	1.013	0.483
Change GFS^b^	−0.59 ± 0.98	0.67 ± 0.95	1.097	0.845	1.425	0.513
Change GFR^c^	−0.99 ± 1.44	−1.11 ± 1.61	1.055	0.901	1.234	0.488

*Note:* For categorical measures, the chi-square statistic is shown along with the *p* values from the Fisher’s exact test. Household income categories (4 = $40–59,000 per year; 5 = $60–99,999).

Abbreviations: SIPS = structured interview for psychosis-risk syndromes; APSS = attenuated psychotic symptoms syndrome; BIPS = brief intermittent psychotic syndrome; GRD = genetic risk and deterioration; BZ = benzodiazepine; SOPS = scale of psychosis-risk symptoms, a part of the SIPS; CDSS = Calgary Depression Scale for Schizophrenia; GAF = global assessment of functioning; GFS = global functioning–social; GFR = global functioning–role; APi = AP inception; nAPi = no AP inception.

a *n* = 79 for APi and 578 for nAPi.

b *n* = 79 for APi and 571 for nAPi.

c *n* = 79 for APi and 579 for nAPi.

d *n* = 79 for APi and 576 for nAPi.

e *n* = 79 for APi and 577 for nAPi.

f *n* = 78 for APi and 579 for nAPi.

g *n* = 78 for APi and 580 for nAPi.

h *n* = 78 for APi and 578 for nAPi.

Separate logistic regressions including each predictor variable along with the effect of study and their interaction were performed using bootstrapping (*n* = 1000) on all variables (univariable analyses in Table 1). When the addition of the interaction term did not significantly improve the model, we removed the interaction term and evaluated the main effect of that variable. In rare cases when the addition of the interaction term did significantly improve the model, we calculated the simple main effects of the predictor for each study. When both studies simple main effects were in the same direction, we considered the overall main effect as interpretable. Similarly, when study simple main effects were in opposite directions but were not statistically significant in either study, we also considered the overall main effect as interpretable. In both of these cases we report the overall main effect from the model step including the interaction term, taking care to code the study alternatives as 0.5 and −0.5. However, when study simple main effects were in opposite directions and were statistically significant within either or both studies, we report the overall main effect of study as noninterpretable. The main effects of study were nonsignificant for all interpretable analyses in Table 1. In view of these findings and of the relatively small number of AP inception participants in each of the NAPLS-2 and -3 cohorts (Figure 1) and of the similar methods employed in the two studies (Addington et al., 2012; Addington et al., 2022), data from NAPLS-2 and -3 were merged. All subsequent analyses were conducted on the combined sample without terms for study.

Categorical variables with a cell size of <5 in the smaller group (*AP inception*) were excluded from the final model due to substantial bias in estimating beta weights (Vittinghoff & McCulloch, 2007). Of the eight remaining significant univariable predictors, two were collinear based on criteria of a Pearson’s *r* value >0.7 (Dormann et al., 2013): SOPS (scale of psychosis-risk symptoms) total score and SOPS general subscale score. Since the SOPS general score contributes to the calculation of the SOPS total and because it better distinguished the *AP inception* from *no AP inception* groups in the univariable analyses, we removed SOPS total from consideration in the final model. The effects of eliminating either SOPS variable on the significance of the other were evaluated in sensitivity analyses.

The number of the remaining variables in Table 1 with significant univariate effects (seven) met the criterion for a minimum of 10 events in the smaller group per predictor (Peduzzi, Concato, Kemper, Holford, & Feinstein, 1996), and we therefore entered those seven predictors into the final logistic regression model (Table 2). The final model calculated individualized predicted probabilities of AP inception for each case. The odds ratios and confidence intervals are reported from the standard logistic regression model, whereas the significance values (*p* values) are reported from the final bootstrap model (*n* = 1000) for both Tables 1 (univariable findings) and 2 (final model).

**Table 2.** Final logistic regression model results

Predictor	Beta	SE of beta	*p*	OR	95% Confidence interval	
					LB	UB
Education years	−0.090	0.035	0.008	0.914	0.848	0.984
Current major depression	0.651	0.304	0.030	1.918	1.078	3.411
Current anxiety disorder	0.259	0.282	0.334	1.295	0.766	2.189
Current benzodiazepine	1.282	0.495	0.003	3.605	1.407	9.241
Any psychotropic	0.273	0.289	0.338	1.313	0.749	2.302
SOPS general	−0.003	0.038	0.914	0.997	0.925	1.074
CDSS total	0.060	0.033	0.061	1.061	0.996	1.131

Overall chi-square = 36.53, df = 7, *p* ≤ 0.001. See Table 1 for abbreviations.

### Exploration of loss of univariable effects in the final model

Finally, in order to understand why variables significant at the univariable level lost significance in the final models, we conducted exploratory logistic regressions removing predictors. These models entered the single variable that was of current interest (because of its lost significance) into the first step and then entered variables that correlated significantly with it (Table 4) one at a time in decreasing order of their correlation. A variable that lost significance in the final model (see Table 2) was considered a proxy predictor (Kraemer, Stice, Kazdin, Offord, & Kupfer, 2001) for another variable when adding the other variable significantly improved the model and led to loss, or further loss, of the original variable’s significance as a predictor.

## Results

### Sample description

A total of 764 CHR-P participants were enrolled in NAPLS-2 (Addington et al., 2012). Of these, 536 (70.2%) had a documented absence of AP medications in their lifetime at baseline (Figure 1), and 28 were excluded due to participating in a randomized clinical trial, leaving 508. Twelve participants who did not meet the SIPS CHR-P criteria were also excluded, leaving 496. An additional 114 were removed because they did not have a full medication record for 6 months after baseline, leaving 382. A total of 49 of the 382 began AP during the first 6 months while CHR-P (12.8%), and 333 did not. Eleven participants first started AP in the first 6 months only after conversion and were considered not AP inception participants while CHR-P.

A total of 710 CHR-P participants were enrolled in NAPLS-3 (Addington et al., 2022). Among these, 453 (63.8%) had documented absence of AP medications in their lifetime at baseline (Figure 1). One case who did not meet the SIPS CHR-P criteria was also excluded, leaving 452. Of these, 175 were removed because they did not have a full medication record for 6 months after baseline, leaving 277. A total of 30 began AP during the first 6 months, while CHR-P (10.8%) and 247 did not. No NAPLS-3 participants first started AP in the first 6 months after conversion.

The combined sample of NAPLS-2 + NAPLS-3 included 79 AP inception participants and 580 participants who did not have AP inception (Figure 1). The antipsychotic medications most commonly prescribed during the first 6 months in the two study samples were risperidone followed by aripiprazole and quetiapine (Table 3). Less commonly prescribed antipsychotics include olanzapine, haloperidol, clozapine, lurasidone, and perphenazine.

**Table 3.** AP medication at inception

AP medication	NAPLS-2 (*n* = 49)	NAPLS-3 (*n* = 30)	Total (*N* = 79)
Risperidone	16 (32.7%)	14 (46.7%)	30 (37.9%)
Aripiprazole	17 (34.7%)	8 (24.2%)	25 (31.6%)
Quetiapine	14 (28.6%)	3 (10%)	17 (21.5%)
Olanzapine	1 (2.0%)	1 (3.3%)	2 (2.5%)
Haloperidol	1 (2.0%)	0 (0%)	1 (1.3%)
Clozapine	0 (0%)	1 (3.3%)	1 (1.3%)
Lurasidone	0 (0%)	1 (3.3%)	1 (1.3%)
Perphenazine	0 (0%)	2 (6.7%)	2 (2.5%)
Total	49	30	79

Table 1 describes the analyzed CHR-P AP inception and no inception samples in terms of baseline demographics, diagnostic groupings, other psychotropic medications, symptoms, and functioning. Exploratory analysis for the participants with missing medication data at 6 months showed that the only difference between the participants with complete medications data and those without complete data was that the participants included in the sample had slightly higher attenuated positive symptom severity (Table S1 data supplement). Table S2 (data supplement) shows the distribution of CHR-P subtypes in the sample.

### Antipsychotic prediction analysis

Logistic regression along with bootstrapping performed on each variable and its interaction with study in Table 1 revealed that seven noncollinear variables (education years, current major depression, current anxiety disorder, current use of benzodiazepines [BZ] and any psychotropic, SOPS general subscale score, and Calgary Depression Scale for Schizophrenia score [CDSS]) were significant univariable predictors (*p* < 0.05).

The final logistic regression model with bootstrapping performed on the remaining seven predictors yielded a highly statistically significant model (χ^2^ = 36.53, df = 7, *p* ≤ 0.001). Figure S1 (data supplement) illustrates prediction effects in the model, showing that higher individual predicted probabilities were associated with higher proportions of actual AP inception. Three variables (fewer education years, current *major depression*, and current benzodiazepine use) emerged as significant independent predictors of AP inception (Table 2). Sensitivity analyses replacing SOPS general subscale with SOPS total produced the same three variables as the only significant predictors.

### Exploration of loss of univariable effects in the final model

The variables significantly predicting AP inception in the univariable model (Table 2) that lost significance in the final model were: current anxiety disorder, any psychotropic medication use, SOPS general subscale score, and CDSS total score. Logistic regression analyses exploring the univariable prediction of AP inception by current anxiety disorder revealed that current anxiety disorder lost significance as a predictor when SOPS general was included and lost further significance when CDSS and again when major depression were then added. Any psychotropic use lost significance as a predictor when current BZ use was included and lost further significance when major depression was then added. SOPS general score lost significance as a predictor when CDSS was added and lost further significance when major depression and again when current BZ were then added. CDSS lost significance as a predictor when major depression was included.

These findings suggest that the variables that lost univariable significance in the final model did so because they were correlated with (Table 4) and, as defined by Kraemer et al. (2001)), ultimately proxies for major depression (all four variables) or proxies for both major depression and current BZ use (SOPS general and any psychotropic use). Current anxiety disorder was also a proxy for CDSS and SOPS general (itself a proxy for CDSS), both of which were proxies for major depression or major depression and current BZ use.

**Table 4.** Correlations among significant univariable predictor variables

	Education years	Current depression	Current anxiety disorder	Benzodiazepines	Any psychotropic	SOPS general	CDSS total
Education years	1	−0.025	−0.022	0.141***	0.027	−0.035	0.051
Current depression	−0.025	1	0.165***	0.003	0.126***	0.388***	0.479***
Current anxiety disorder	−0.022	0.165***	1	0.046	0.029	0.294***	0.175***
Benzodiazepines	0.141***	0.003	0.046	1	0.363***	0.104***	0.057
Any psychotropic	0.027	0.126***	0.029	0.363***	1	0.061	0.068
SOPS general	−0.035	0.388***	0.294***	0.104**	0.061	1	0.551***
CDSS total	0.051	0.479***	0.175***	0.057	0.068	0.551***	1

^*^p < 0.05, ^**^p < 0.01, ^***^ *p* < 0.001. *N* varied from 654 to 657 depending on missing data. See Table 1 for abbreviations.

## Discussion

The principal findings of this analyses are: (1) baseline demographic, diagnostic, medication, symptom, and functioning measures in previously AP-naive CHR-P participants were predictive of AP inception over the next 6 months; (2) presence of current major depression, previous prescription of BZ, and fewer years of education significantly and independently predicted AP inception; and (3) contrary to our expectations, severity of attenuated positive symptoms was not a significant predictor of AP inception.

We speculate that major depression in CHR-P may predict AP inception in part because prescribers for CHR-P patients who have current comorbid depression may be using the AP to augment a suboptimal antidepressant effect. Major depression is a frequent comorbid diagnosis in patients at CHR-P (Albert, Tomassi, Maina, & Tosato, 2018; Paolo Fusar-Poli, Nelson, Valmaggia, Yung, & McGuire, 2012; Rosen, Miller, D’Andrea, McGlashan, & Woods, 2006; Solmi et al., 2023), including in the current sample (Table 1), and antidepressants are commonly used in CHR-P patients (Fusar-Poli et al., 2015; Raballo, Poletti, & Preti, 2023; Woods et al., 2013). The effectiveness of antidepressants in CHR-P patients, however, may often be suboptimal (McGorry et al., 2023). In major depression, when antidepressants are less than fully effective, augmenting AD prescription with AP is common practice (Rhee, Mohamed, & Rosenheck, 2018). As reported in 2023, four APs were FDA approved for augmentation in major depression (aripiprazole, brexpiprazole, olanzapine, and quetiapine) (Kishimoto, Hagi, Kurokawa, Kane, & Correll, 2023), three of which were used in the current sample (Table 3). Risperidone, the most commonly started AP in this sample (Table 3), is not FDA approved for augmentation in major depression, but evidence that it may be effective (Owenby, Brown, & Brown, 2011) could nevertheless be reflected in off-label use.

Interestingly, current major depression but not current antidepressant use (AD) was a significant and independent predictor of AP inception because ADs are also used for anxiety disorders. Major depression may also be a stronger predictor than AD use because use of ADs in combination with AP may be less common in anxiety disorders than in major depression. As reported in 2020, only one AP is approved by FDA for anxiety disorder (Garakani et al., 2020), a first-generation AP (trifluoperazine) not used in the current sample (Table 3).

We speculate that current prescription of BZ medication predicted AP inception because it may be a marker of higher symptom severity prior to baseline that of course could not be measured. In our data, current BZ prescription at baseline was associated with higher SOPS general symptom scores (Table 4) despite the medication, raising the possibility that the baseline scores reflect only partial response which prescribers may have attempted to address using AP. The possibility that BZ prescription reflects a severity marker is supported by a recent report that BZ exposure in CHR-P patients was associated with increased attenuated positive symptom severity as compared to the BZ-unexposed group (Livingston et al., 2024). Another possibility is that BZ prescription may reflect greater access to providers who commonly employ a wider range of psychotropics.

Fewer years of education emerged as a significant and independent predictor of AP inception in CHR-P patients. Fewer years of education was highly correlated with age in the combined samples (*r* = 0.755, *p* < 0.001), but age was not itself a significant predictor of AP inception even at the univariable level in our sample (Table 1). One paper evaluating data from the 2005 US Medical Expenditure Panel Survey reports that fewer years of patient education was associated with a greater likelihood of AP prescription (Wang & Farley, 2013). In that paper, the effect of education became nonsignificant when adjusting for socioeconomic and healthcare access measures, suggesting that years of education was a proxy for those measures. However, in this study another socioeconomic variable (household income) was not a significant univariable predictor of AP inception (Table 1).

Severity of attenuated positive symptoms may not have been a significant predictor of AP inception for several reasons. First, patients often do not spontaneously disclose attenuated positive symptoms to providers because of the risk of stigma and discrimination. Concerns that CHR-P will be stigmatized arise due to conflation by third parties of CHR-P with other psychotic disorders and also due to patients internalizing stigma about their symptoms and diagnosis (Zhang, McConnell, Carter, & Pugh, 2023). In addition, a qualitative study evaluating young men in Norway suggests that CHR-P patients were not disclosing their symptoms due to other factors beyond stigma. Many patients noticed a change in their well-being but did not identify the change due to potential mental illness. Others did not recognize that anything was changing or had difficulty describing the change. Some participants also did not differentiate normal fluctuations of mood from positive symptoms (Åmlid, Carlsson, Bjørnestad, Joa, & Hegelstad, 2024).

Yet another reason why severity of attenuated positive symptoms may not have been a significant predictor of AP inception is that many community prescribers may not be adept in eliciting attenuated positive symptoms or in detecting them even when patients do disclose them. A qualitative study evaluating Southwest England general practitioners’ (GPs’) views and experiences in identifying the CHR-P syndrome suggests that some GPs were not familiar with the concept of being at clinical high risk and also perceived that they may not have the right skill set to identify this population. Some GPs also mentioned that if a patient already met the criteria for a more common mental illness such as depression or anxiety, they would not always screen for psychotic symptoms (Strelchuk, Wiles, Derrick, Zammit, & Turner, 2021).

This article may be the first to report on predictors of AP *start* in CHR-P patients, using predictors measured before the lifetime onset of AP medication. Previous studies of predictors of AP *use* have focused on use of APs already present at baseline and on predictors measured at the same baseline time point after AP start (Pelizza, Leuci, et al., 2024; Zeng et al., 2022; Zhang et al., 2022). Even if predicting AP use at baseline is considered a proxy for predicting AP inception, these studies may incorporate bias into that prediction, for two reasons. First, predictors measured at baseline in the group taking AP medication at baseline may have been affected by the AP treatment that occurred prior to baseline. Second, the group *not* receiving AP currently at baseline may contain a mixture of patients who are AP naive with those who *had* previously started on AP (and then discontinued before baseline). Both of these biases could make it more difficult to detect symptoms and functioning as predictors, potentially by including patients with improved symptoms and functioning in the user group and/or by including patients with worsened symptoms and functioning in the nonuser group. Despite these concerns, Zeng et al. and Pelizza et al. both detected higher positive symptom severity and greater drop in GAF score as significant predictors of AP use at baseline in both univariable and multivariable models (Zeng et al., 2022) and in their univariable model (Pelizza, Leuci, et al., 2024). These differences from our findings could relate to one or more of several factors: the larger sample size in Zeng et al., greater recognition of CHR-P symptoms among prescribers at the single Shanghai site (Zeng et al., 2022), greater recognition of CHR-P symptoms in Parma due to their specialized clinical protocol (Pelizza, Leuci, et al., 2024), or the use in Parma of only univariable models. Other differences from our findings included that Pelizza et al. reported education years as nonsignificant, which may relate to their smaller sample size, and older age as a significant predictor of AP use at baseline, which we speculate could potentially have been affected by adult versus pediatric prescriber mix. Zeng et al. may have not detected years of education as significant in their multivariable model due to multicollinearity with age. Neither study could conclude that BZ use or current major depression were predictors of AP use at baseline because those variables were not reported.

## Limitations

The NAPLS studies were not specifically designed for the prediction of the AP inception in CHR-P youth. As a consequence, data were missing for medications over the 6 months following baseline in 289 (30.5%) of 948 participants, leading to a smaller sample size and hence decreased statistical power. Comparisons of the samples with and without missing medications data, however, suggested that the missing data did not lead to a biased sample, with the exception of lower attenuated positive symptom scores in the participants with missing medication data. The impact of this difference is difficult to judge because the participants with the missing medication data could not be assigned to *AP inception* versus *no inception* groups. A second limitation is that we did not have the opportunity to inquire about the therapeutic rationales of the prescribers who prescribed the antipsychotics to the CHR-P patients or whether they had detected attenuated positive symptoms. Similarly, NAPLS-2 and NAPLS-3 did not collect any data about the professional specialty of prescribers or whether they were affiliated with a CHR-P specialty clinic. Furthermore, the studies were based in the United States and Canada; thus, our findings may not generalize to settings with different prescribing guidelines and healthcare systems. Future studies should be designed specifically to examine the prescribers’ rationales for use of AP in CHR-P youth and should collect larger samples from more diverse settings. A third limitation is that we could not investigate the effects of negative symptoms or current GFR in the combined sample due to inconsistent effects between the NAPLS studies. A fourth limitation is that the degree of distress due to the symptoms was not captured by the SOPS score used to determine attenuated positive symptom severity. If possible, future studies should determine whether distress due to attenuated positive symptoms predicts AP inception. Fifth, our analyses were limited to predicting AP inception over the next 6 months after baseline instead of over a longer time frame. Sixth, one of the AP inception cases was on clozapine (Table 3), which was surprising given that clozapine is usually reserved for refractory illness. However, we confirmed that the medication record was complete with no errors and that results of the final model would be virtually identical if this unusual case were removed. Finally, the study lacks external validation which limits our ability to assess the generalizability of the results.

## Supporting information

Mukhtar et al. supplementary material Mukhtar et al. supplementary material

## Data Availability

The data collected for the NAPLS-3 study are available from the US National Institute of Mental Health Data Archive, including individual participant data and a data dictionary defining each field in the set, will be made available to others. Requests for data collected for the NAPLS-2 may be submitted to the corresponding author.

## Supplementary material

The supplementary material for this article can be found at http://doi.org/10.1017/S0033291725101372.

## Summary

This study is the first to collect prediction data before AP start in patients at clinical high risk for psychosis and to determine baseline characteristics for the prediction of AP initiation, a design that minimizes confounding effects of previous treatment. Our findings shed light on why antipsychotics are prescribed to CHR-P youth in spite of the current clinical guidelines. At least some AP use in CHR-P appears to be intended as augmentation of antidepressant treatment for comorbid major depression. Some prescribers may not be aware of attenuated positive symptoms since their severity did not significantly predict AP inception. Our findings will also inform the design of future longitudinal CHR-P research studies regarding the likelihood of new AP prescription during follow-up.
