# Supplementary material for: Prediction of antipsychotic medication inception in antipsychotic-naive youth at clinical high risk for psychosis
Source: Psychol Med. 2025 Aug 22;55:e241. doi: 10.1017/S0033291725101372 (PMC12404330; doi:10.1017/S0033291725101372)
Supplement: Mukhtar et al. supplementary material [file S0033291725101372sup001.docx]

**Data Supplement**

**Table S1.**

| Measure | Complete  Medication Data  (N=659) | Missing Medication Data  (N=289) | X^2^ or t (df) | p |
| --- | --- | --- | --- | --- |
| Demographics | | | | |
| Age | 18.48±4.26 | 18.28±4.17 | 0.678(946) | 0.498 |
| Male gender | 365(55.4%) | 143(44.6%) | 2.817 (1) | 0.093 |
| Caucasian^m^ | 362(54.9%) | 149(51.7%) | 0.824(1) | 0.364 |
| Latino^n^ | 124(18.9%) | 60(20.8%) | 0.490(1) | 0.484 |
| Household income^j^ | 4.73±1.91 | 4.80±2.05 | -0.470(507.5) | 0.639 |
| Education years^k^ | 11.31±3.25 | 11.13±3.26 | 0.810(944) | 0.418 |
| Diagnostic groupings | | | | |
| COPS APSS | 639(97%) | 278(96.2%) | 0.378(1) | 0.539 |
| COPS BIPS | 11(1.7%) | 4(1.4%) | 0.105(1) | 0.746 |
| COPS GRD | 43(6.5%) | 22(7.6%) | 2.671 (1) | 0.263 |
| Current major depression^o^ | 275(41.7%) | 127(45.7%) | 1.248(1) | 0.264 |
| Current bipolar disorder^p^ | 36(5.5%) | 12(4.3%) | 0.535(1) | 0.464 |
| Current anxiety disorder^o^ | 353(53.6%) | 148(53.2%) | 0.008(1) | 0.927 |
| Baseline Medications | | | | |
| Antidepressant | 146(22.2%) | 51(17.6%) | 2.480(1) | 0.115 |
| Mood stabilizer | 9(1.4%) | 5(1.7%) | 0.183 (1) | 0.669 |
| Psychostimulant | 38(5.8%) | 25(8.7%) | 2.694(1) | 0.101 |
| Benzodiazepine | 33(5%) | 9(3.1%) | 1.701(1) | 0.192 |
| NonBZ anxiolytic | 3(0%) | 2(0.3%) | 0.215(1) | 0.643 |
| Any psychotropic | 188(28.5%) | 71(24.6%) | 1.587(1) | 0.208 |
| Symptoms | | | | |
| SOPS Total^b^ | 37.78±12.22 | 36.35±13.16 | 1.593(929) | 0.111 |
| SOPS Positive^a^ | 12.38±3.61 | 11.80±3.69 | 2.271(945) | 0.023 |
| SOPS Negative^c^ | 11.52±6.03 | 10.90±6.18 | 1.411 (930) | 0.159 |
| SOPS Disorganized^d^ | 4.94±3.05 | 4.72±3.01 | 0.993(931) | 0.321 |
| SOPS General^c^ | 8.99±4.25 | 8.90±4.54 | 0.301(930) | 0.763 |
| CDSS total^e^ | 5.84±4.59 | 5.95±4.68 | -0.340(919) | 0.734 |
| Functioning | | | | |
| Current GAF^f^ | 50.94±11.15 | 52.46±11.95 | -1.890(943) | 0.059 |
| Current GSF^g^ | 6.41±1.52 | 6.47±1.52 | -0.558(942) | 0.577 |
| Current GRF^h^ | 6.31±2.16 | 6.44±2.05 | -0.849(941) | 0.396 |
| Change in Functioning | | | | |
| Change GAF^i^ | -8.94±11.05 | -8.40±12.11 | -0.666(940) | 0.506 |
| Change GSF^g^ | -0.66±0.95 | -0.74±0.99 | 1.148(942) | 0.251 |
| Change GRF^h^ | -1.10±1.59 | -1.16±1.55 | 0.567(941) | 0.571 |

For categorical measures the X^2^ statistic is shown along with p-values from the Fisher’s exact test.

Household income categories (4=$40-59,000 per year; 5=$60-99,999).

^a^ n = 658 for complete data and 289 for missing data

^b^ n = 655 for complete data and 276 for missing data

^c^ n = 656 for complete data and 276 for missing data

^d^ n = 657 for complete data and 276 for missing data

^e^ n = 657 for complete data and 264 for missing data

^f^ n = 658 for complete data and 287 for missing data

^g^ n = 658 for complete data and 286 for missing data

^h^ n = 657 for complete data and 286 for missing data

^i^ n = 656 for complete data and 286 for missing data

^j^ n = 650 for complete data and 284 for missing data

^k^ n = 658 for complete data and 288 for missing data

^l^ n = 658 for complete data and 287 for missing data

^m^ n = 659 for complete data and 288 for missing data

^n^ n = 657 for complete data and 288 for missing data

^o^ n = 659 for complete data and 278 for missing data

^p^ n = 658 for complete data and 278 for missing data

**Table S2. Distribution of Clinical High Risk Syndromes AP inception.**

| Syndromes | Any APSS | Any BIPS | Any GRD |
| --- | --- | --- | --- |
| One syndrome only | 606/639 (94.8%) | 2/11(18.18%) | 18/43(41.86%) |
| +APSS only | - | 8/11(72.73%) | 24/43(55.81%) |
| +BIPS only | 8/639(1.25%) | - | 0/43(0%) |
| +GRD only | 24/639(3.76%) | 0/11(0%) | - |
| +APSS and BIPS | - | - | 1/43(2.33%) |
| +APSS and GRD | - | 1/11(9.09%) | - |
| +BIPS and GRD | 1/639(0.16%) | - | - |
| Total Complete Data | 639/659 (96.9%) | 11/659(1.67%) | 43/659(6.53%) |

**Figure S1: Predicted vs actual Probability of AP Inception.**


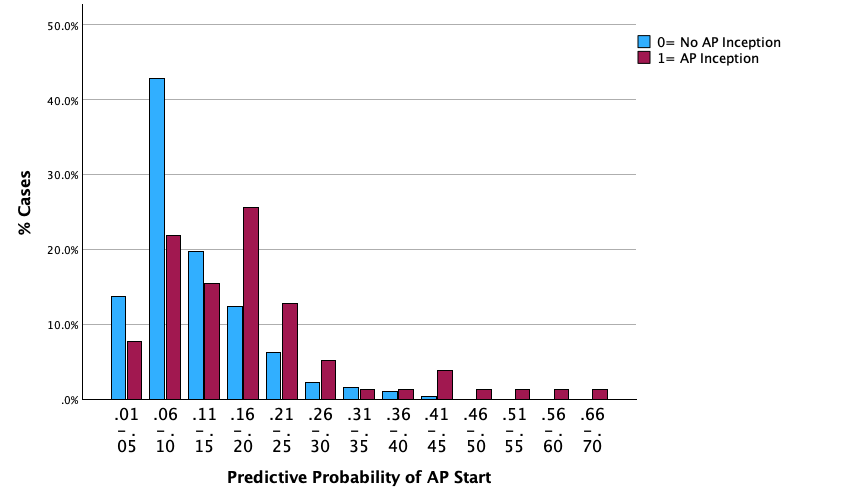


**Figure Legend:**

The number of cases refers to the number of participants. The chart displays the actual probability of AP start vs predicted probability of AP start in inception and no inception cases from the final model.
